# Characterization of the acetylation of cyclooxygenase-isozymes and targeted lipidomics of eicosanoids in serum and colon cancer cells by the new aspirin formulation IP1867B *versus* aspirin *in vitro*

**DOI:** 10.3389/fphar.2022.1070277

**Published:** 2022-12-14

**Authors:** Ulrika Hofling, Stefania Tacconelli, Annalisa Contursi, Annalisa Bruno, Matteo Mucci, Patrizia Ballerini, Simon Cohen, Paola Patrignani

**Affiliations:** ^1^ Center for Advanced Studies and Technology (CAST), “G. D’Annunzio” University, Chieti, Italy; ^2^ Department of Neuroscience, Imaging and Clinical Science, Medical School, “G. D’Annunzio” University, Chieti, Italy; ^3^ Department of Innovative Technologies in Medicine and Dentistry, “G. D’Annunzio” University, Chieti, Italy; ^4^ Innovate Pharmaceuticals, Manchester, United Kingdom

**Keywords:** aspirin, IP1867B, platelet, serum, HCA7 cells, cyclooxygenases, HETEs, lipidomics

## Abstract

**Background:** Aspirin(acetylsalicylic acid, ASA) is recommended for the secondary prevention of atherothrombotic events and has shown anticancer effects. The current enteric-coated drug formulation may reduce aspirin bioavailability. Liquid formulations could improve aspirin pharmacokinetics and pharmacodynamics. IP1867B is a liquid-aspirin formulation that combines three ingredients, ASA/triacetin/saccharin.

**Methods:** ASA and IP1867B(L-ASA) were assessed in human serum(obtained by allowing to clot human whole blood at 37 °C for 1h), washed platelets, and colonic adenocarcinoma HCA7 cells on eicosanoid generation and COX-isozyme acetylation at Serine529 and 516 by LC-MS/MS.

**Results:** In serum, ASA and L-ASA acted by selectively affecting COX-1-derived eicosanoids, including thromboxane(TX)B_2_. L-ASA was more potent in inhibiting serum TXB_2_, a known biomarker of aspirin antiplatelet effect, than ASA. However, ASA and L-ASA were equipotent to acetylate COX-1 in washed platelets and COX-2 in HCA7 cells. In HCA7 cells, ASA and L-ASA acted by inhibiting prostaglandin(PG)E_2_(the most abundant prostanoid) and TXB_2_ biosynthesis. In the presence of a high arachidonic acid concentration(100 μM), 15R-hydroxyeicosatetraenoic acid(HETE) was generated at baseline by cancer cell COX-2 and was only slightly enhanced by supratherapeutic concentrations of ASA(1 mM). In whole blood and HCA7 cells treated with ASA or L-ASA, 15-epi-lipoxin(LX)A_4_ were undetectable.

**Conclusion:** IP1867B was more potent in affecting serum TXB_2_ generation than ASA. The relevance of this finding deserves evaluation *in vivo* in humans. In cancer cells, ASA and IP1867B acted by inhibiting PGE_2_ and TXB_2_ generation *via* the acetylation of COX-2. ASA and IP867B at clinically relevant concentrations did not substantially induce the biosynthesis of 15R-HETE and 15-epi-LXA_4_.

## 1 Introduction

Cyclooxygenase (COX)-1 and -2 play a role in different physiological settings and disease processes mainly because of the striking differences in their tissue expression and regulation (Crofford, 1997; Dubois et al., 1998; Simmons et al., 2004). However, the structures and catalytic functions of COX-1 and COX-2 are almost entirely identical. The enzymes possess two separated but linked active sites, cyclooxygenase and peroxidase (Simmons et al., 2004). The cyclooxygenase active site catalyzes the *bis*-dioxygenation of arachidonic acid (AA) to prostaglandin (PG)G_2,_ which is reduced to PGH_2_ by the peroxidase active site (Rouzer & Marnett, 2020). Then, the downstream synthases, expressed in a tissue-specific manner, transform PGH_2_ in the different prostanoids. COX-isozymes can produce small amounts of 11- and 15-hydroperoxyeicosatetraenoic acids (HpETEs), which are subsequently converted by the peroxidase activity of these enzymes to the corresponding hydroxyeicosatetraenoic acids (HETEs) (Thuresson et al., 2000; Harman et al., 2004). 11-HETE generated by COX-1 and COX-2 is exclusively in the R-configuration (Hawkins & Brash, 1987; Baer et al., 1991). 15-HETE derived from COX-2 is also in the R configuration, whereas COX-1 gives rise to both 15S-HETE and 15R-HETE (Thuresson et al., 2000; Powell and Rokach, 2015).

COX-1 displays the characteristics of a “housekeeping” gene and is constitutively expressed in almost all tissues (Crofford, 1997). Platelets express only COX-1 and generate mainly thromboxane(TX)A_2_, a potent platelet activator and vasoconstrictor, which undergoes rapid non-enzymatic hydrolysis to biologically inactive TXB_2_ (Dubois et al., 1998; Patrignani & Patrono 2015). In contrast, COX-2 is the product of an “immediate-early” gene that is rapidly induced and tightly regulated (FitzGerald, 2003; Capone et al., 2007). Under basal conditions, COX-2 expression is highly restricted to the vasculature and brain; however, COX-2 is dramatically upregulated during inflammation (Crofford, 1997). In cancer cells, COX-2 is persistently expressed due to posttranscriptional mechanisms leading to the stabilization of mRNA (Young & Dixon 2010). Several lines of evidence support the central role of COX-2-dependent PGE_2_ in inflammation and tumorigenesis (Wang & DuBois, 2010, 2013). TXA_2_ can also exert a protumorigenic action. It has been shown that the knockdown of TBXA2R (the gene encoding TXA_2_ receptor, TP) or TBXAS1(the gene encoding the downstream synthase TXS, which catalyzes the conversion of PGH_2_ to TXA_2_) in human colorectal cancer cells resulted in fewer colonies being formed in soft agar than in control cells (Li et al., 2015).

Recently, the analyses of randomized clinical trials (RCTs) performed to assess the efficacy and safety of aspirin (acetylsalicylic acid, ASA), in the prevention of cardiovascular disease showed a significant reduction in the incidence and mortality of cancer, mainly in the colorectum (Thun et al., 2002; Patrignani & Patrono, 2016). The mechanism of aspirin’s anticancer effects is still under debate.

Since the drug was also effective at the low doses recommended for the secondary prevention of cardiovascular disease (Baigent et al., 2009), it has been hypothesized that the inhibition of platelet function was involved in colorectal cancer (CRC) chemoprevention (Patrignani & Patrono, 2016; Patrignani & Patrono 2018; Dovizio et al., 2020). In fact, low-dose aspirin causes irreversible inhibition of platelet COX-1 (Roth & Majerus, 1975; Patrignani et al., 1982) *via* the acetylation of Serine529 within the cyclooxygenase active site (Patrignani et al., 2014; Patrignani & Patrono 2015). However, prostanoids generated in the gastrointestinal mucosa are also involved in cancer development and progression. Low-dose aspirin can incompletely affect COX-1-dependent prostanoids in the intestinal mucosa (Patrignani et al., 2017) due to low concentrations detected in the systemic circulation and aspirin’s short half-life.

Aspirin can also acetylate COX-2 at Serine516 (Lecomte et al., 1996; Tacconelli et al., 2020b). This effect is associated with the efficient inhibition of prostanoid formation by COX-2; however, acetylated COX-2 seems to be able to generate 15R-HETE, which has been reported to become the major AA metabolite produced under these conditions (Lecomte et al., 1996). It has been proposed that aspirin-acetylated COX-2 is also responsible for the formation of aspirin-triggered lipoxins (LXs), such as 15-epi-LXA_4_ (Claria & Serhan, 1995; Chiang et al., 2004) *via* transcellular conversion by cells expressing 5-lipoxygenase (LOX).

The current formulation of low-dose aspirin is enteric-coated (EC), which was developed to reduce stomach damage. However, the coating reduces aspirin absorption in some people leading to reduced bioavailability (Maree et al., 2005; Grosser et al., 2013). Recently, novel formulations of aspirin have been developed to improve the drug’s systemic bioavailability, leading to improved capacity to affect extraplatelet sources of prostanoids possibly implicated in multiple disease processes (Bliden et al., 2016; Angiolillo et al., 2022; Rade et al., 2022). Thus, extended-release ASA formulations can lead to sustained blood levels of ASA (Bliden et al., 2016). A liquid aspirin formulation (L-ASA) is in clinical development. It is named IP1867B and combines three ingredients ASA, triacetin, and saccharin.

This study aimed to characterize ASA and IP1867B (L-ASA) in human platelets and the human colon cancer cell line HCA-7 on the capacity to acetylate COX-1 and COX-2 at Serine529 and Serine516, respectively. Moreover, we evaluated the concentration-response of the two compounds in affecting the generation of targeted lipidomics of eicosanoids (prostanoids, HETEs, and 15-epi-LXA_4_) in serum and HCA-7 cells (expressing COX-2, 5-LOX, and 15-LOX-1, while COX-1 and platelet-type 12-LOX were undetectable) by chiral liquid chromatography-mass spectrometry (LC-MS/MS).

## 2 Materials and methods

### 2.1 Materials

Acetonitrile (ACN), water (LC-MS grade), formic acid (FA), n-hexane, methanol, acetic acid, and isopropanol were from Carlo Erba Reagenti, Milan, Italy. Standards of TXB_2_, PGE_2_, HETEs, and 15-epi-LXA_4_ and their deuterated forms were from Cayman Chemical (Ann Arbor, Michigan, United States). ECL Western Blotting Detection Reagents were from GE Healthcare (Milan, Italy). Acetylsalicylic acid (ASA, aspirin), dimethyl sulfoxide (DMSO), ethanol (ETOH), bovine serum albumin (BSA), NaCl, KCl, MgCl_2_, CaCl_2_, glucose, citric acid, citrate-dextrose solution (ACD), Triton X-100, phenylmethylsulfonyl Fuoride (PMSF), the β-actin monoclonal antibody (cat# A5316), dextran from Leuconostoc spp, Hystopaque 1,077, Dulbecco′s Modified Eagle′s Medium (DMEM), Penicillin-Streptomycin, Fetal Bovine Serum (FBS), endoproteinase Glu-C Sequencing Grade (Glu-C), arachidonic acid (AA), ammonium bicarbonate (NH_4_HCO_3_), dithiothreitol (DTT) were from Sigma Aldrich, Milan, Italy. The columns (Aeris 1.7 μm PEPTIDE XB-C18 100 Å, 100 × 2.1 mm and Lux^®^ 3 μmAmylose-1, 150 mm × 3.0 mm) were from Phenomenex, Torrance, CA, United States. The Bradford protein assay, the Laemmli Buffer, β-Mercaptoethanol, the PVDF membrane, and the non-fat milk for immunoblot were from Bio-Rad, Milan, Italy. The anti-platelet type 12-LOX polyclonal antibody (item#Ab211506) and the anti-15-LOX-1 polyclonal antibody (item #Ab80221) were from Abcam, Cambridge, UK; the anti-β-actin monoclonal antibody was from Santa Cruz Biotechnology (item#sc8432); the anti-5-LOX polyclonal antibody was a gift from Dr. Dieter Steinhilber (Goethe University, Frankfurt, Germany). Colon cancer cell line HCA7 colony 29 (HCA7) was from the European Collection of Cell Cultures (ECC, Salisbury, UK). Trypsin Gold (Mass Spectrometry Grade) and ProteaseMAX™ Surfactant, Trypsin Enhancer were from Promega (Wisconsin, United States). The light standard peptides (unacetylated and acetylated) of COX-1 and COX-2 [for COX-1: IGAPFSLK and IGAPFS(Ace)LK; for COX-2: VGAPFSLK and VGAPFS(Ace)LK] and the corresponding AQUA peptides, which were incorporated with stable isotopes ^13^C and ^15^N (Patrignani et al., 2014; Tacconelli et al., 2020b) were synthesized by Thermo Fisher Scientific (Waltham, MA, United States) (>97% purity). NuPAGE^®^ Novex 7% Tris-Acetate Gel, RIPA buffer, GelCode Blue Stain Reagent, NuPAGE LDS sample buffer, EDTA-free proteases inhibitors were from Thermo Fisher Scientific (Waltham). L-ASA (IP1867B) and triacetin/saccharin (called diluent) were a gift from Innovate Pharmaceuticals, Manchester, United Kingdom.

### 2.2 Effects of ASA and L-ASA on eicosanoid lipidomics of human whole blood assay *in vitro*

Peripheral venous blood samples were drawn from healthy volunteers (n = 10, 23–50 years) when they had not taken any non-steroidal antiinflammatory drug (NSAID) during the 2 weeks preceding the study. This study was carried out following the recommendations of the Declaration of Helsinki after approval by the local Ethics Committee of “G. d' Annunzio” University of Chieti-Pescara (#254), and informed consent was obtained from each subject.

ASA (0.005–150 mM) was dissolved in DMSO; IP1867B (L-ASA 0.005–150 mM) was dissolved in triacetin (glycerin triacetate) and saccharin (named diluent) (kindly provided by Innovate Pharmaceuticals, United Kingdom); then 2-µL aliquots of the vehicles or the different solutions of ASA or L-ASA were pipetted directly into glass test tubes to give final concentrations of 0.01–300 µM (for ASA) and 0.01–300 µM (for L-ASA) in 1 ml of blood. One-ml aliquots of whole blood were immediately transferred into glass tubes and allowed to clot at 37 °C for 1 h. After incubation, serum was immediately separated by centrifugation (1560 g for 10 min at 4°C) and stored at –80°C until assayed for TXB_2_, which reflects platelet COX-1 activity (Patrono et al., 1980) by immunoassay (Cayman Chemical, item#501020) and LC-MS/MS (Patrignani et al., 2014; Tacconelli et al., 2020a). In serum, 12R-HETE, 12S-HETE, 15R-HETE, 15S-HETE, 5R-HETE, 5S-HETE, 8R-HETE, 8S-HETE, 15-epi-LXA_4,_ and PGE_2_ were also assessed by LC-MS/MS as previously reported (Tacconelli et al., 2020a) and briefly described below. The impact of DMSO and triacetin-saccharin on the lipidomics of serum eicosanoids at baseline (in the absence of vehicles) was also evaluated.

### 2.3 Effects of L-ASA and ASA on TXB_2_ biosynthesis and COX-1 acetylation in washed human platelets

Human platelets were freshly isolated from leukocyte concentrates obtained from Centro Trasfusionale, at Renzetti Hospital, Lanciano, Italy, as previously described (Dovizio et al., 2012; Dovizio et al., 2013a). Washed platelets presented only 0.38 ± 0.29% (mean ± SEM) of leukocyte contamination as assessed by flow cytometry (Grande et al., 2019). Platelets (1 × 10^8^) suspended in a final volume of 0.5 ml of HEPES buffer [containing 5 mM HEPES, 137 mM NaCl, 2 mM KCl, 1 mM MgCl_2_, 12 mM NaHCO_3_, 0.3 mM NaH_2_PO_4_, and 5.5 mM glucose, pH 7.4, 3.5 mg/ml BSA] were treated with different concentrations of ASA (0.01–300 µM or DMSO) or L-ASA (0.01–300 µM or diluent) for 30 min at room temperature. Then, CaCl_2_ 2 mM and MgSO_4_ 1 mM were added for 2 min at room temperature, and then platelets were stimulated with AA (10 μM) for 30 min at 37°C. The reaction was stopped by keeping the tubes at 4°C before the centrifugation at 2200 *g* for 5 min (at 4°C) to separate the platelet pellet from the supernatant, which was collected and centrifuged at 10,000 g for 5 min at 4°C. The supernatant was stored at −80°C. TXB_2_ levels were assessed in the supernatants by a specific immunoassay (Patrignani et al., 2014). In the same experimental conditions, the % acetylation of COX-1 was assessed in 2.0 × 10^7^ platelets treated with ASA, L-ASA, DMSO, or diluent by LC-MS/MS as previously described (Patrignani et al., 2014). The cells were lysed and subjected to in-gel digestion using trypsin and GluC (Drapeau et al., 1972; Brown and Wold, 1973). The samples containing the peptides obtained by the proteolysis were dried by vacuum centrifugation and stored at -80 °C until analyzed for the concentrations of endogenously formed IGAPFSLK, its acetylated form, and the AQUA peptides by LC-MS/MS (Patrignani et al., 2014).

### 2.4 Effects of ASA and L-ASA on COX-2 acetylation and eicosanoid biosynthesis in HCA7 cells

HCA7 cells (3.0 × 10^6^) were seeded and cultured in DMEM supplemented with 10% FBS and 1% penicillin-streptomycin for 48 h, then the medium was changed to DMEM supplemented with 0.5% FBS, and 1% penicillin-streptomycin. HCA-7 cells were assessed for COX-1 and COX-2 levels by using LC-MS/MS, as previously described (Patrignani et al., 2014; Tacconelli et al., 2020b). 5-LOX, 15-LOX-1, and platelet type 12-LOX were assessed by Western blot as reported below.

The cells were treated with increasing concentrations of ASA (1–1000 µM), L-ASA (1–1000 µM), or with DMSO or diluent for 60 min at 37 °C. The cells were lysed and subjected to in-gel digestion using trypsin and GluC (Drapeau et al., 1972; Brown and Wold, 1973; Tacconelli et al., 2020b). The samples containing the peptides obtained by the proteolysis were dried by vacuum centrifugation and stored at -80 °C until analyzed for the concentrations of endogenously formed VGAPFSLK, its acetylated form, and the AQUA peptides by LC-MS/MS (Tacconelli et al., 2020b). The effect of ASA or L-ASA on the activity of COX-2 was evaluated by preincubating the cells (1 × 10^6^), cultured as reported above, with increasing concentrations of ASA (1–1,000 µM), L-ASA (1–1,000 µM), DMSO or diluent for 30 min at 37°C; finally, cells were incubated with AA (0.5, 10 and 100 µM) for 30 min at 37°C. At the end of the incubation, conditioned media were harvested and centrifuged at 10,000 g for 5 min to discard cell debris, and the supernatant was stored at -80 °C until assayed for the levels of eicosanoids by using LC-MS/MS (Tacconelli et al., 2020a). The generation of eicosanoids (12R-HETE, 12S-HETE, 15R-HETE, 15S-HETE, 5R-HETE, 5S-HETE, 8R-HETE, 8S-HETE, 15-epi-LXA_4_, TXB_2_ and PGE_2_) was assessed by using LC-MS/MS. Samples were extracted by using a liquid-liquid extraction (Maskrey et al., 2007; Tacconelli et al., 2020a). Briefly, to 1 ml of the sample, 2.5 ml of a mixture of acetic acid/isopropanol/hexane (2:20:30, v/v/v) and internal standards (d_8_-12S-HETE, d_8_-15S-HETE, d_8_-5S-HETE, d_4_TXB_2_, and d_4_-PGE_2_; at the final concentration of 5 ng/ml) were added. The extraction was performed by adding 5 ml of n-hexane. Then, the samples were centrifuged at 1500 g at 4°C for 5 min. The dried hexane phases were stored at −80°C until LC-MS/MS analysis. Before analysis, dried lipids were resuspended in 200 μL of methanol and analyzed by LC-MS/MS as previously described (Tacconelli et al., 2020a). The LC-MS/MS system consisted of ACQUITY UPLC I-Class/Xevo TQ-S micro IVD System (Waters) equipped with a Z-Spray ESI source under negative ionization conditions. Deuterated and non-deuterated standards (from Cayman Chemical) were analyzed in MS/MS mode to examine the collision-induced fragmentation spectrum. In Supplementary Table, the list of the molecular ions and fragments monitored for each eicosanoid is shown. Separation of 12R-HETE, 12S-HETE, 15R-HETE, 15S-HETE, 5R-HETE, 5S-HETE, 8R-HETE, 8S-HETE, 15-epi-LXA_4_, TXB_2_ and PGE_2_ was performed using a chiral chromatographic column (Lux 3  μm Amylose-1, 150 mm × 3.0 mm; Phenomenex, Torrance, CA, United States) eluting a 20-min gradient of 50–100% solvent B (60% methanol, 40% ACN, 0.1% glacial acetic acid) and solvent A (75% water, 25% ACN, 0.1% glacial acetic acid): 50% solvent B for 5 min; 50–60% solvent B for 4 min; 60–80% solvent B for 2 min; 80–90% solvent B for 2 min; 90–100% solvent B for 1 min, 100% solvent B for 2 min and 50% of solvent B from 17 to 20 min with a flow rate of 0.2 ml/min). The linear standard curves were obtained by adding constant amounts of internal standards to eight different concentrations of each analyte (0.01–500 ng/ml), then the calibration curves were constructed by linear regression of the ratio of the peak areas of the analytes to the areas of the corresponding internal standards. For 8R- and 8S-HETE and 15-epi-LXA_4_, we used d_8_-12S-HETE as the internal standard because the deuterated forms are not commercially available. The eicosanoid concentrations were calculated by interpolation from the calculated regression lines. The peptide peak areas were extracted and analyzed by using MassLynx software (Waters, United Kingdom). The data were normalized to sample volume and expressed as ng/ml. The detection limit of quantification of each eicosanoid was 10 pg/ml.

### 2.5 Western blot analysis

The protein levels of 5-LOX, 15-LOX-1, and platelet-type-12-LOX in HCA7 lysates from HCA7 were determined by Western blot analysis, as previously described (Dovizio et al., 2013b). Briefly, an aliquot of the sample was loaded onto 9% Sodium Dodecyl Sulphate PolyAcrylamide Gel Electrophoresis (SDS-PAGE), transferred to polyvinylidene difluoride (PVDF) membrane (GE Healthcare, Milan, Italy) and blocked with a solution of 5% non-fat milk in tris-buffered saline-0.1% Tween-20 (TBS-Tween-20). The membranes were incubated overnight with primary antibodies diluted in TBS-Tween-20, platelet-type12-LOX (dilution 1:1,000), 5-LOX (dilution 1:50) or 15-LOX-1 (dilution 1:1,000), and β-actin (dilution 1:1,000). Then, the membranes were washed in TBS-Tween-20 and incubated with the secondary antibodies. Quantifying the optical density (OD) of different specific bands was calculated using Alliance 1 D software (UVITEC, Cambridge) and normalized to the OD of β-actin.

### 2.6 Statistical analysis

The data have been reported as mean ± SD or SEM. The statistical analysis was performed using GraphPad Prism software (version 9.00 for Windows; GraphPad, San Diego, CA). The values of *p* < 0.05 were considered statistically significant. The concentration-response curves were obtained using PRISM (GraphPad, version 9.00 for Windows). The IC_50_ (compound concentration to inhibit by 50% eicosanoid biosynthesis) or EC_50_ (compound concentration to cause 50% of maximal effect) and 95% confidence interval (CI) values of the sigmoidal concentration-response data were obtained by GraphPad Prism software.

## 3 Results

### 3.1 Effects of ASA and L-ASA on eicosanoid generation in human whole blood

**FIGURE 1:**
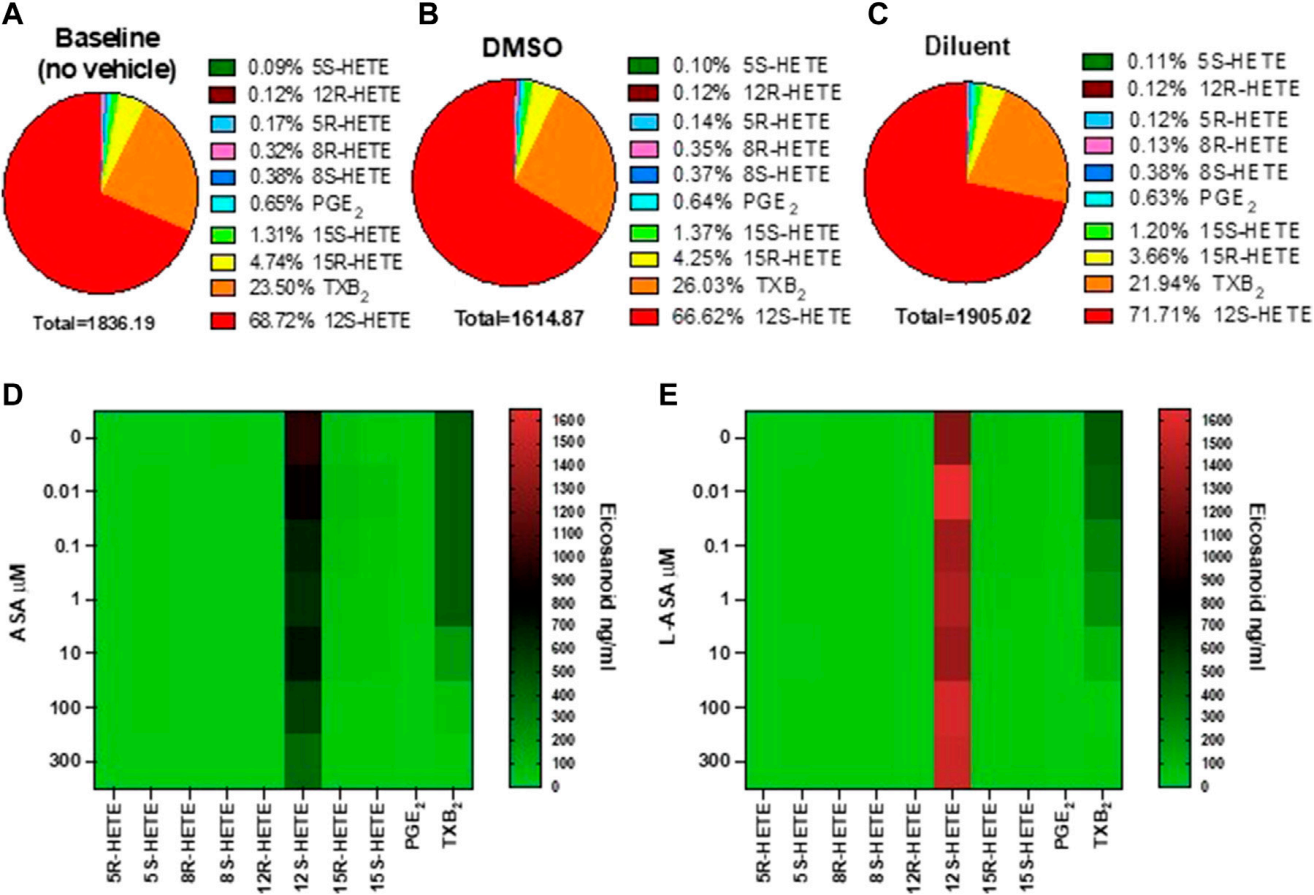
Baseline values and the effects of ASA or L-ASA on eicosanoids of human serum generated by targeted chiral lipidomics analysis using LC-MS/MS. **(A–C)** Pie chart reporting the generation of eicosanoids in serum samples obtained in human whole blood allowed to clot for 1 h at 37 °C without vehicles **(A)** and in the presence of DMSO (ASA vehicle) **(B)** or diluent (triacetin and saccharin, the constituents of L-ASA) **(C)**; data are the percentage of each eicosanoid *versus* the total concentration of all eicosanoids obtained by the mean of 6 separate experiments. **(D,E)** Heat map of the effect of ASA and L-ASA (0.01–300 μM), respectively, on eicosanoid (mean values of three separate experiments) biosynthesis in serum, respectively. As described in the Methods Section, eicosanoid levels were measured by LC-MS/MS.

We have previously reported the targeted chiral LC-MS/MS analysis of human serum obtained by peripheral whole blood allowed to clot for 1 h at 37°C (Tacconelli et al., 2020a). Under these conditions, thrombin is generated endogenously, and activating the PARs (protease-activated receptors) induces the release of AA from membrane phospholipids which can be transformed enzymatically and non-enzymatically into prostanoids and HETEs. The influence of DMSO (used as a vehicle in ASA experiments) and triacetin/saccharin (used in L-ASA experiments, called diluent) on serum eicosanoid generation at baseline (i.e., in the absence of vehicles) was assessed (Figures 1A–C and Supplementary Figure S1). In serum, 12S-HETE and TXB_2_ were the major eicosanoids (67.72% and 23.5%, respectively). They are mainly of platelet origin (Tacconelli et al., 2020a). PGE_2_ and other HETEs were minor products. Prostanoids and low levels of 15S- and 15R-HETE originate from COX-1 activity since COX-2 is not expressed in clotting whole blood (Patrignani et al., 1999). 5S-HETE, the product of the 5-LOX, was only 0.09% of all eicosanoids. Moreover, non-enzymatic oxidation of AA led to tiny levels of 12R-HETE, 8R-HETE, 8S-HETE, and 5R-HETE (i.e., 0.99%), as reported before (Mazaleuskaya et al., 2018; Tacconelli et al., 2020a).

DMSO did not significantly affect the generation of these eicosanoids (Supplementary Figure S1). In the presence of the diluent, a slight, significant increase of 12S-HETE and a decrease of 8R-HETE (a marginal product) was detected *versus* DMSO (Supplementary Figure S1). However, the concentration of the other eicosanoids in serum, including TXB_2_, was not significantly influenced by DMSO or the diluent (Supplementary Figure one).

The heatmap representation of the effects of ASA and L-ASA on serum eicosanoids is reported in Figures 1D,E, respectively. ASA and L-ASA caused a concentration-dependent reduction of TXB_2_, while 12S-HETE was substantially unaffected (Supplementary Figures S2A,B).

**FIGURE 2:**
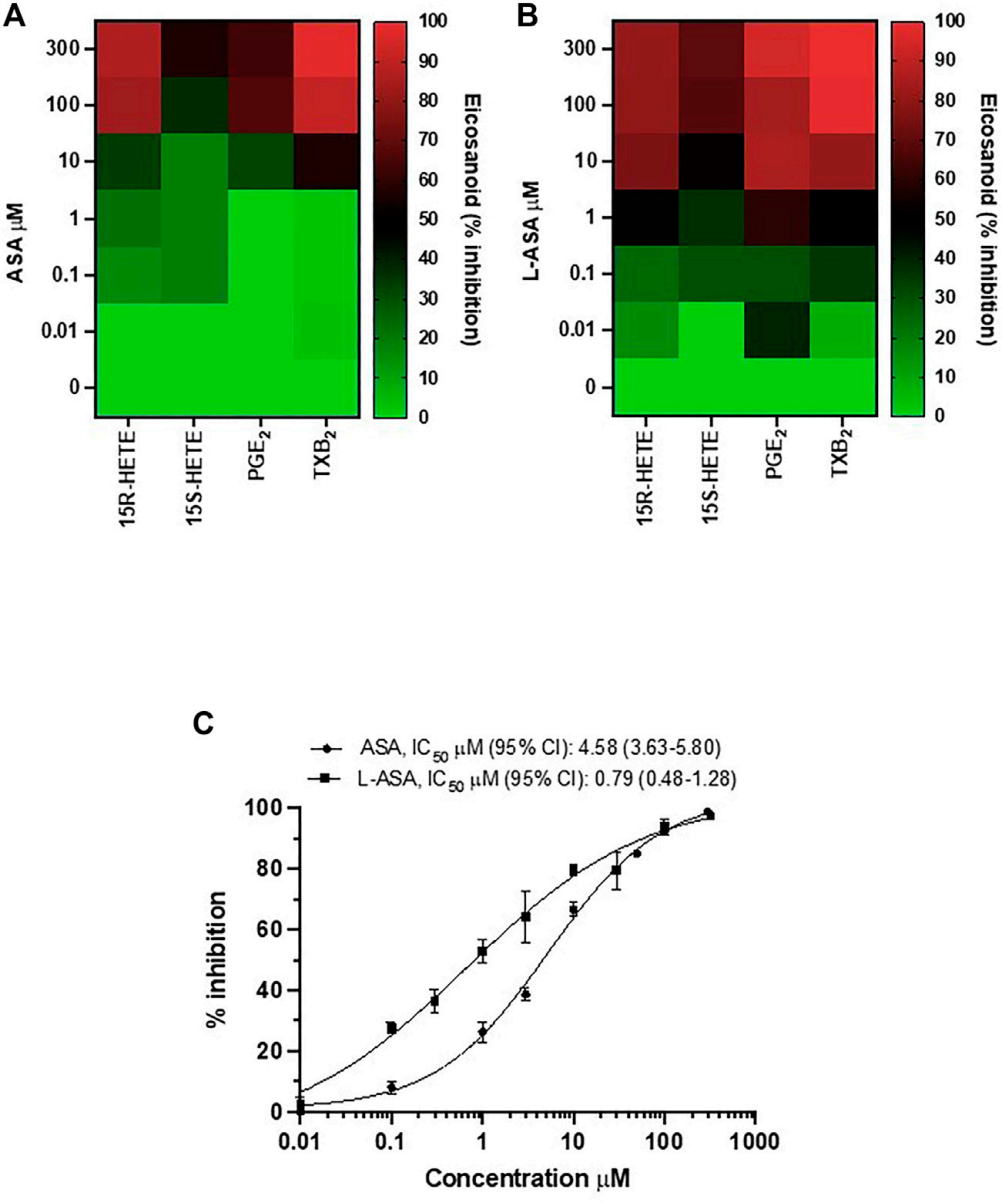
Comparison of the effects of Aspirin and L-ASA on eicosanoids generated from COX-1 in whole blood. Aliquots (1 ml) of whole blood were collected from healthy volunteers and incubated with increasing concentrations of ASA (0.01–300 μM) or L-ASA (0.01–300 µM) or their vehicles (DMSO or diluent) for 1 h at 37°C. Serum was obtained by centrifugation, and 15S-HETE and 15R-HETE, PGE_2_, and TXB_2_ levels were measured by LC-MS/MS. **(A,B)** Heat map of the effect of ASA and L-ASA on eicosanoids biosynthesis in serum (mean values of three separate experiments); results are reported as % of inhibition from baseline values, in the presence of DMSO or diluent, respectively. **(C)** Concentration-response curve for inhibition of serum TXB_2_ by ASA and L-ASA using a validated and specific immunoassay; results are reported as mean ± SD (n = 4–7) of % inhibition from baseline values, in the presence of DMSO or diluent, respectively; concentration-response curves were fitted, and IC_50_ with 95% Confidence Interval (CI) values were reported.

The heatmap of the inhibitory effect of the COX-1 products, TXA_2_, PGE_2_, 15S- and 15R-HETE by ASA and L-ASA is also shown in Figures 2A,B, respectively. ASA and L-ASA caused a concentration-dependent reduction of the four eicosanoids. These results are also represented as concentration-response sigmoidal curves for the inhibition of eicosanoid production by ASA and L-ASA (Supplementary Figures S2A,B). ASA and L-ASA showed concentration-dependent inhibitory effects on TXB_2_, PGE_2_, and 15R-HETEs. Variable and less pronounced effects on the generation of the other HETEs was found (Supplementary Figures S2A,B). L-ASA was associated with higher inhibitory potency on whole blood COX-1-derived eicosanoids than ASA (Figures 2A,B, Supplementary Figures S2A,B, Supplementary Table S2).

Due to the relevant role of TXA_2_ in platelet function, we compared the concentration-response curves of ASA and L-ASA for inhibition of serum TXB_2_ in seven different separate experiments; we show that L-ASA inhibited serum TXB_2_ with IC_50_ values which were 5.8-fold lower than those found with ASA, in a statistically significant fashion. However, at 100–300 μM, ASA and L-ASA caused a comparable and almost virtually complete inhibition of serum TXB_2_ generation (Figure 2C).

As reported above, the diluent of L-ASA alone did not affect serum TXB_2_ levels *versus* DMSO and baseline (without any vehicle) (Supplementary Figure S1), suggesting that it did not contribute to the higher potency of L-ASA *versus* aspirin.

We also assessed 15-epi-LXA_4_ in serum at baseline and after ASA or L-ASA, but it was undetectable (<10 pg/ml) (not shown).

### 3.2 Effects of ASA and L-ASA on TXB_2_ biosynthesis and COX-1 acetylation on washed human platelets

**FIGURE 3:**
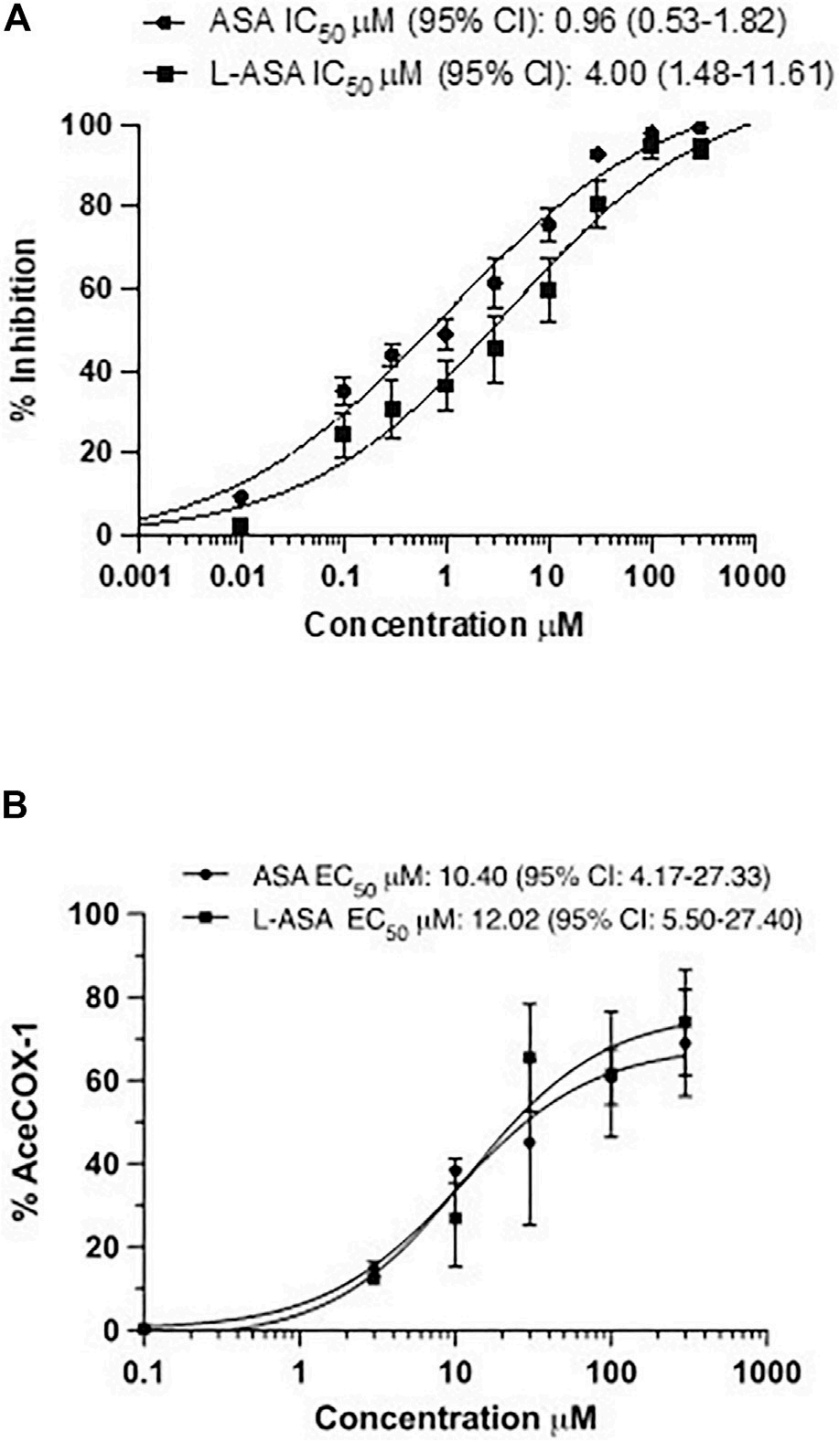
Effects of ASA and L-ASA on the biosynthesis of TXB_2_ and the extent of acetylation of COX-1 at Serine529 in washed human platelets. **(A)**Washed platelet suspensions (0.5 ml containing 1 × 10^8^ cells) were incubated with DMSO or diluent or ASA or L-ASA for 30 min at room temperature; then CaCl_2_ 2 mM and MgSO_4_ 1 mM were added for 2 min at room temperature, and platelets were stimulated with AA 10 μM for 30 min at 37 °C; finally, platelet suspensions were centrifuged, and supernatants were collected to assess TXB_2_ levels by immunoassay (validated by comparison with LC-MS/MS); results are reported as % inhibition from baseline values (mean ± SD, n = 4–6). **(B)** Washed human platelet suspensions were incubated for 60 min with DMSO or diluent or increasing concentrations of ASA or L-ASA at 37°C; then, platelets were lysed, and the quantification of acetylated and unacetylated COX-1 at Serine529 was assessed by LC-MS/MS, as described in materials and methods; the percentage of COX-1 acetylation to total COX-1 was calculated (%AceCOX-1). The data are reported as mean ± SD (n = 3 separate experiments). Concentration-response curves were fitted, and IC_50_ or EC_50_ values with 95% Confidence Interval (CI) values were reported.

We characterized the potency and maximal inhibitory effects of ASA and L-ASA on TXB_2_ production in washed human platelets stimulated with 10 μM of AA (Figure 3A). Under these experimental conditions, the IC_50_ values for ASA and L-ASA were not significantly different (Figure 3A). The two compounds caused comparable concentration-response curves for the acetylation of platelet COX-1 (Figure 3B). The maximal % acetylation of COX-1 by ASA and L-ASA obtained at 100 μM (60.8 ± 6.7 and 61.4 ± 15%, respectively) was slightly enhanced at the higher concentration of 300 μM (69.0 ± 13.0 and 74.4 ± 13.0%, respectively), but the differences were not statistically significant. These data show that in isolated platelets, ASA and L-ASA affect platelet COX-1 activity and TXA_2_ biosynthesis with comparable potency and maximal efficacy. The baseline values of TXB_2_ in the presence of DMSO or diluent were similar (1,008 ± 68 and 999 ± 143 ng/ml, respectively).

Altogether these data show that in isolated platelets, the two compounds have similar pharmacodynamics, i.e., inhibiting COX-1-dependent TXA_2_ generation *via* the acetylation of COX-1.

### 3.3 Biosynthesis of eicosanoids by HCA7 cells in response to exogenous AA

The human colonic adenocarcinoma cell line HCA7 was studied because it expresses COX-2, while COX-1 is undetectable. This finding was previously reported using Western blot (Tacconelli et al., 2020b); here, we confirm it using LC-MS/MS (Supplementary Figure S3A). The cells also expressed 15-LOX-1 and 5-LOX, while platelet-type12-LOX was undetectable (Western blot analysis) (Supplementary Figure S3B).

**FIGURE 4:**
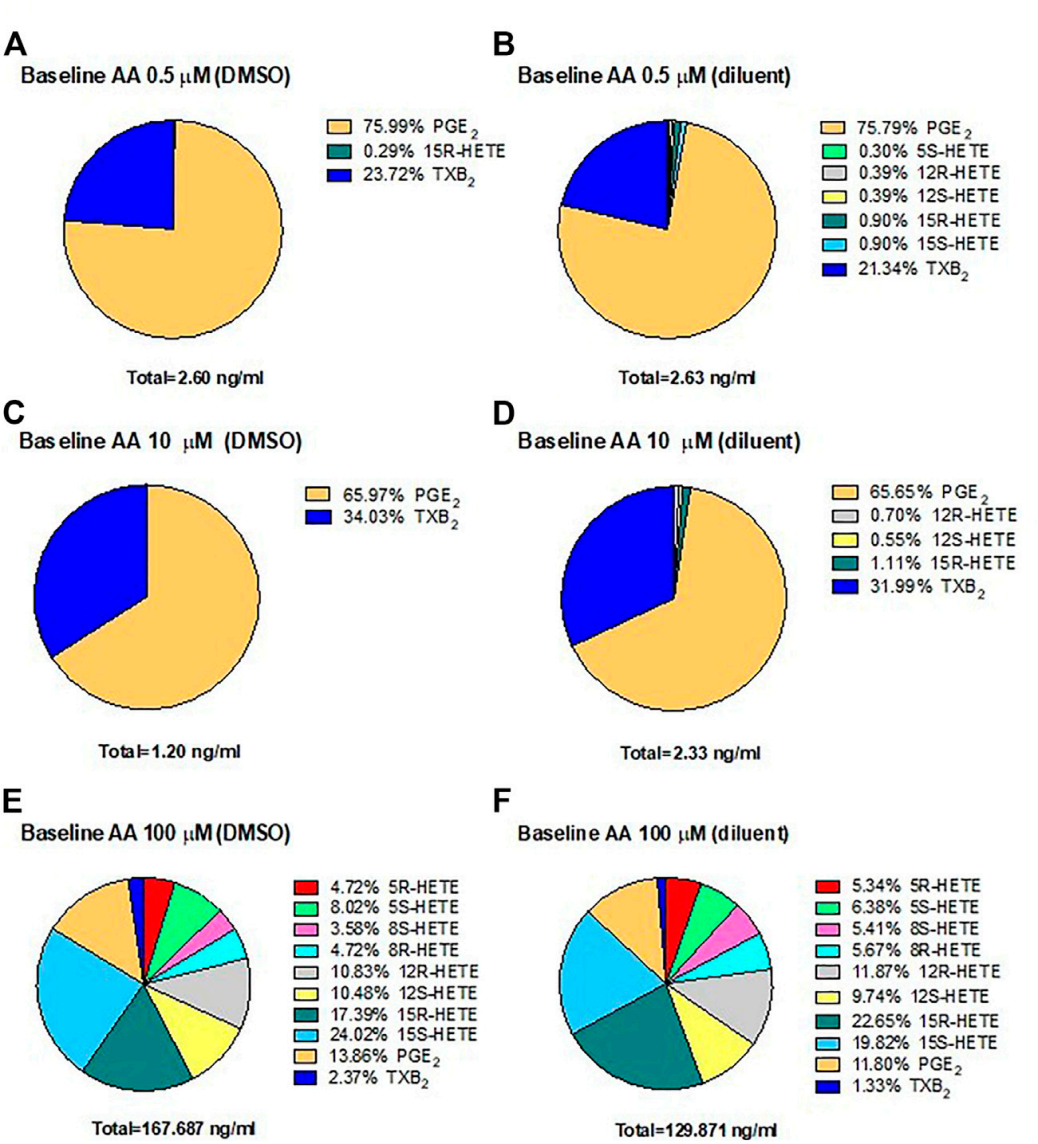
Biosynthesis of eicosanoids by human colon cancer HCA7 cells in response to physiological or high concentrations of exogenous AA in the presence of DMSO or diluent (triacetin and saccharin) by targeted chiral lipidomics analysis using LC-MS/MS. HCA7 cells (1 × 10^6^) were treated with DMSO or diluent for 30 min, and then AA 0.5 **(A,B)**, 10 **(C,D)**, or 100 μM **(E,F)** was added, and the incubation was continued for further 30 min; then, prostanoids and HETEs were assessed in the conditioned medium by LC-MS/MS. Pie charts reporting the generation of eicosanoids in cancer cells from 4 separate experiments; data are shown as the percentage of each eicosanoid *versus* the total concentration of all eicosanoids produced in the presence of DMSO (ASA vehicle) or diluent (triacetin, saccharin, constituents of L-ASA).

In HCA7 cells, we assessed the generation of prostanoids, HETEs, and 15-epi-LXA_4_ using LC-MS/MS in response to physiological concentrations of AA, 0.5 and 10 μM, or the high AA concentration of 100 μM. The biosynthesis of eicosanoids was assessed in the presence of DMSO (Figures 4A,C,E) or the diluent (Figures 4B,D,F). In response to AA 0.5 μM (Figures 4A,B) or 10 μM (Figures 4C,D), PGE_2_ represented 76%, and TXB_2_ was ∼20–30% of all eicosanoids generated. At 100 μM of AA (Figures 4E,F), 15S-HETE, 15R-HETE, and PGE_2_ were the primary products. However, TXB_2_, 5S-HETE, 12S-HETE, and non-enzymatically formed HETEs (5R-HETE, 8R-HETE, 8S-HETE, and 12R-HETE) were detected. 15-epi-LXA_4_ was undetectable. (<10 pg/ml) (not shown). Comparable results were obtained in the presence of DMSO (vehicle of ASA) or triacetin/saccharin (diluent; vehicle of L-ASA) (Figures 4E,F).

These results suggest that PGE_2_ is the main eicosanoid generated in HCA7 cells in response to 0.5 and 10 μM of AA, i.e., at concentrations closer to those released endogenously from cellular membrane phospholipids.

### 3.4 Effects of ASA and L-ASA on the acetylation of COX-2 in HCA7 cells

**FIGURE 5:**
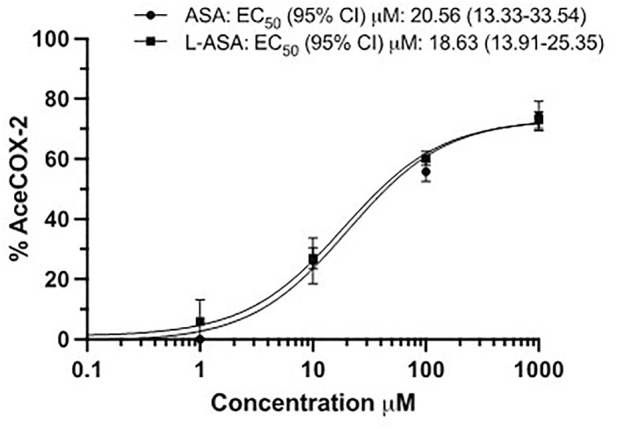
The extent of COX-2 acetylation at Serine516 in HCA-7 cells by ASA and L-ASA. HCA-7 cells (3.0 × 10^6^) were treated with DMSO, the diluent or increasing concentrations of ASA or L-ASA for 60 min at 37 °C. The cells were lysed, and the quantification of acetylated and unacetylated COX-2 was performed after protein digestion by LC-MS/MS as described in Materials and Methods. The percentage of COX-2 acetylation to total COX-2 was calculated (%AceCOX-2). Results are shown as mean ± SD, n = 3–4. Concentration-response curves were fitted, and IC_50_ or EC_50_ values with 95% Confidence Interval (CI) values are reported.

As shown in Figure 5, ASA and L-ASA caused concentration-dependent acetylation of cancer cell COX-2 with comparable potency. However, the maximal % acetylation of COX-2 by ASA and L-ASA was obtained at 1,000 μM (74 ± 5 and 73 ± 3%, respectively). These values resulted significantly (*p* < 0.01) higher than those found at 100 μM (56 ± 3 and 60 ± 4%, respectively). The two compounds’ % acetylation of COX-2 at 100 μM was not significantly different from the % acetylation of platelet COX-1 (Figure 3B).

### 3.5 Effects of ASA and L-ASA on eicosanoid generation in response to AA in HCA7 cells

**FIGURE 6:**
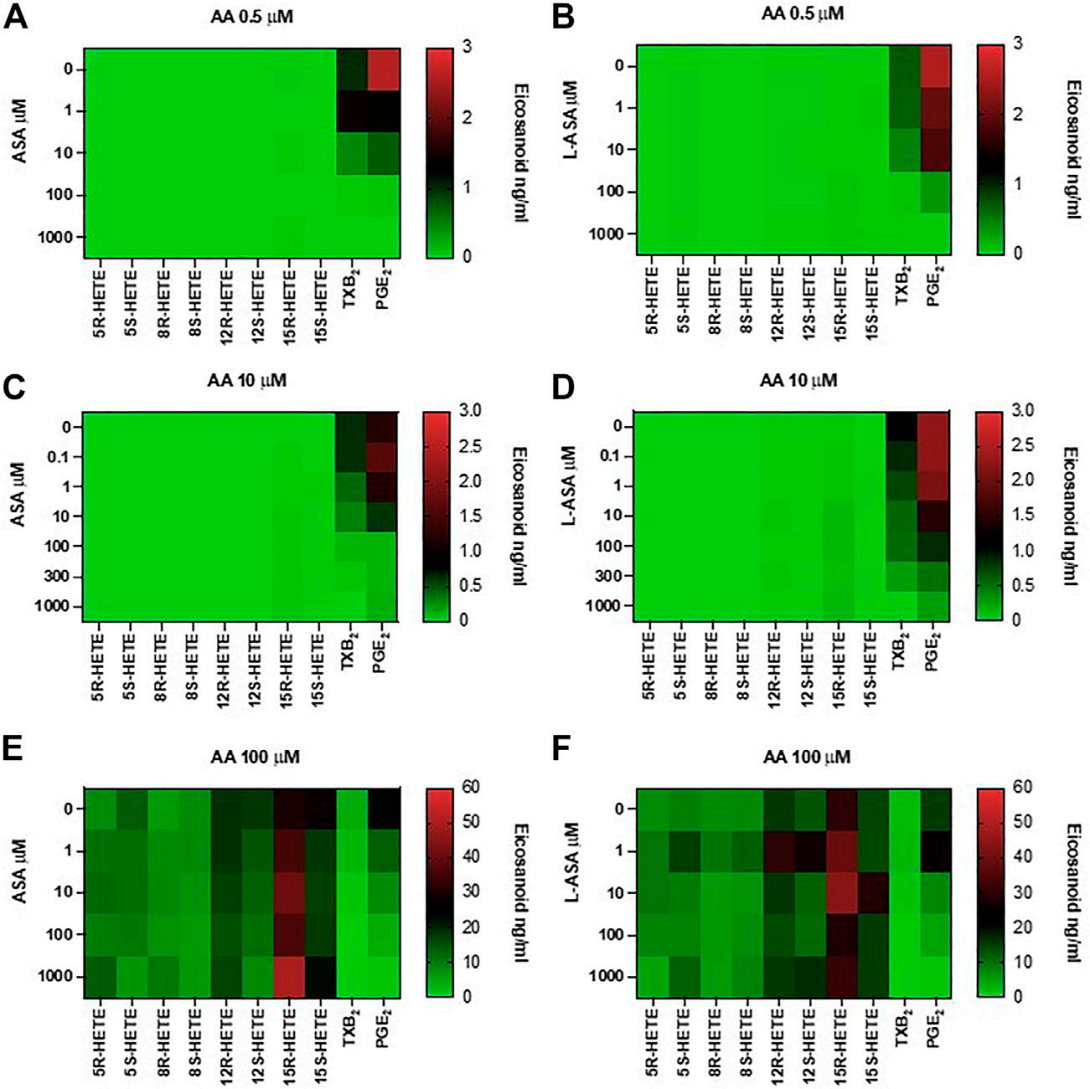
Effects of ASA and L-ASA on eicosanoid generation in response to physiological or high concentrations of AA in HCA7 cells. HCA-7 cells (1 × 10^6^) were treated with DMSO or diluent or increasing concentrations of ASA or L-ASA for 30 min at 37°C, then AA 0.5 **(A,B)**, 10 **(C,D)**, or 100 μM **(E,F)** was added, and the incubation was continued for further 30 min. Prostanoids and HETEs were assessed in the conditioned medium by LC-MS/MS. A heat map of the effect of ASA and L-ASA on eicosanoids biosynthesis (mean values of 4 separate experiments) is shown; results are reported as ng/ml.

In Figure 6, heat maps display the concentration-dependent effects of ASA (panels A, C, E) and L-ASA (panels B, D, F) on eicosanoid generation at 0.5, 10, and 100 μM of AA, respectively.

ASA and L-ASA caused a concentration-dependent reduction of PGE_2_ and TXB_2_ production at all AA concentrations (Figures 6A,C,E, and Figures 6B,D,F, respectively). AA 100 μM was associated with the production of HETEs, and ASA and L-ASA caused marginal and variable effects (Figures 6E,F, respectively). 15-epi-LXA_4_ was undetectable in all experimental conditions (not shown).

**FIGURE 7:**
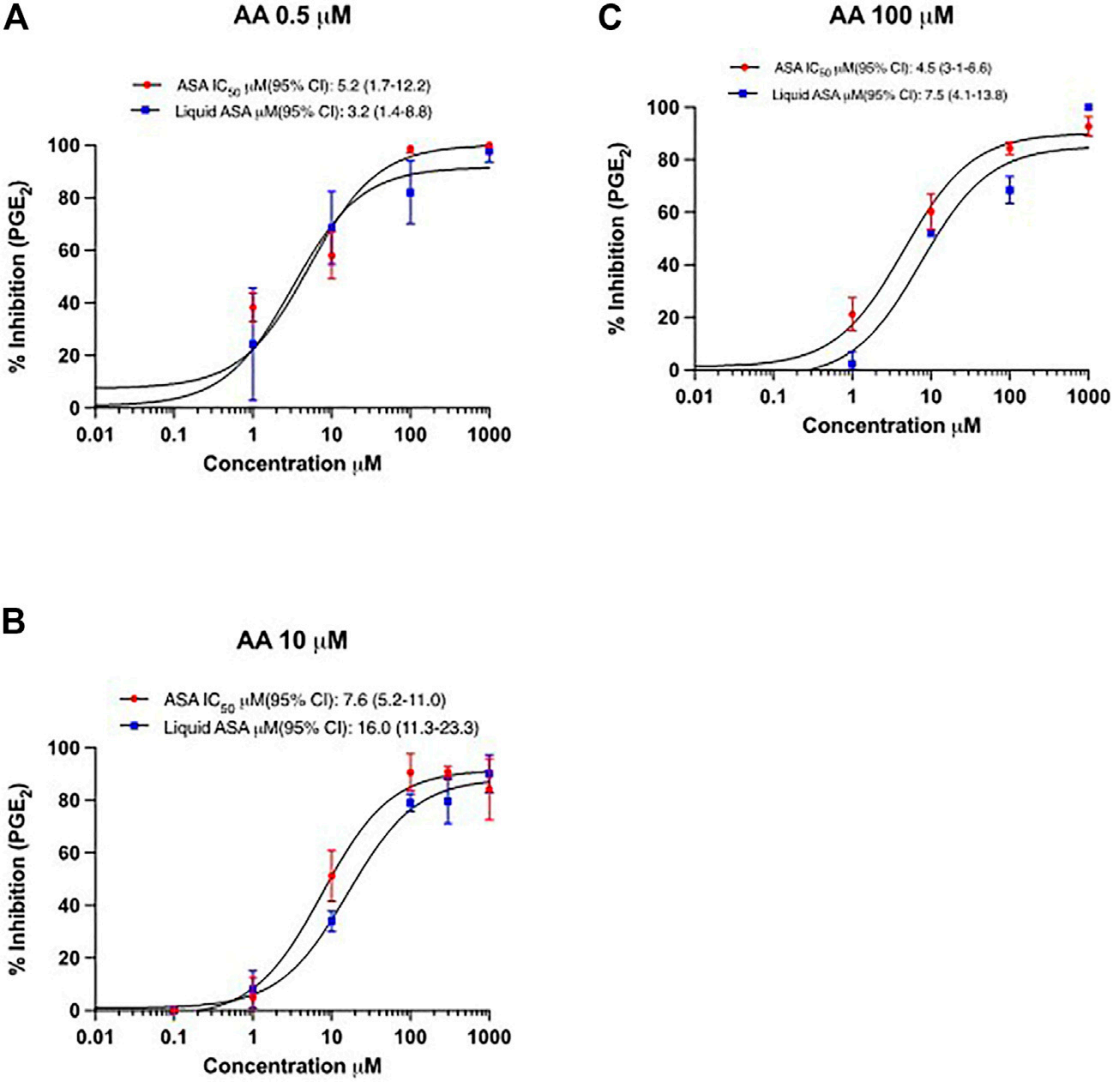
Concentration-response curves for inhibition of PGE_2_ in HCA7 cells in response to different concentrations of AA by ASA and L-ASA. The biosynthesis of PGE_2_ in HCA7 cells was evaluated by preincubating the cells (1 ×10^6^) with vehicles (DMSO or diluent) or increasing concentrations of ASA or L-ASA for 30 min at 37°C; then, cells were incubated with AA 0.5 **(A)**, 10 **(B)** or 100 µM **(C)** for 30 min at 37°C and the levels of PGE_2_ were assessed in the medium by LC-MS/MS. Data (% of inhibition from baseline with DMSO or diluent) are reported as mean ± SD, n = 4. Concentration-response curves were fitted, and IC_50_ values with their 95% CI values are shown.

The inhibitory effect on PGE_2_ biosynthesis in HCA7 cells stimulated with the different concentrations of AA by the increasing concentrations of ASA or L-ASA is reported in Figures 7A–C. The IC_50_ values of PGE_2_ inhibition were not significantly different for ASA and L-ASA at each AA concentration (Figures 7A–C and Supplementary Table S3). Comparable results were obtained for TXB_2_ (Supplementary Figures S4A-C and Supplementary Table S3). These findings show the irreversible nature of COX-2 inhibition by the two compounds. If the interaction of the compounds with COX-2 was reversible, we would have seen a decrease in potency as a function of increased AA concentrations.

**FIGURE 8:**
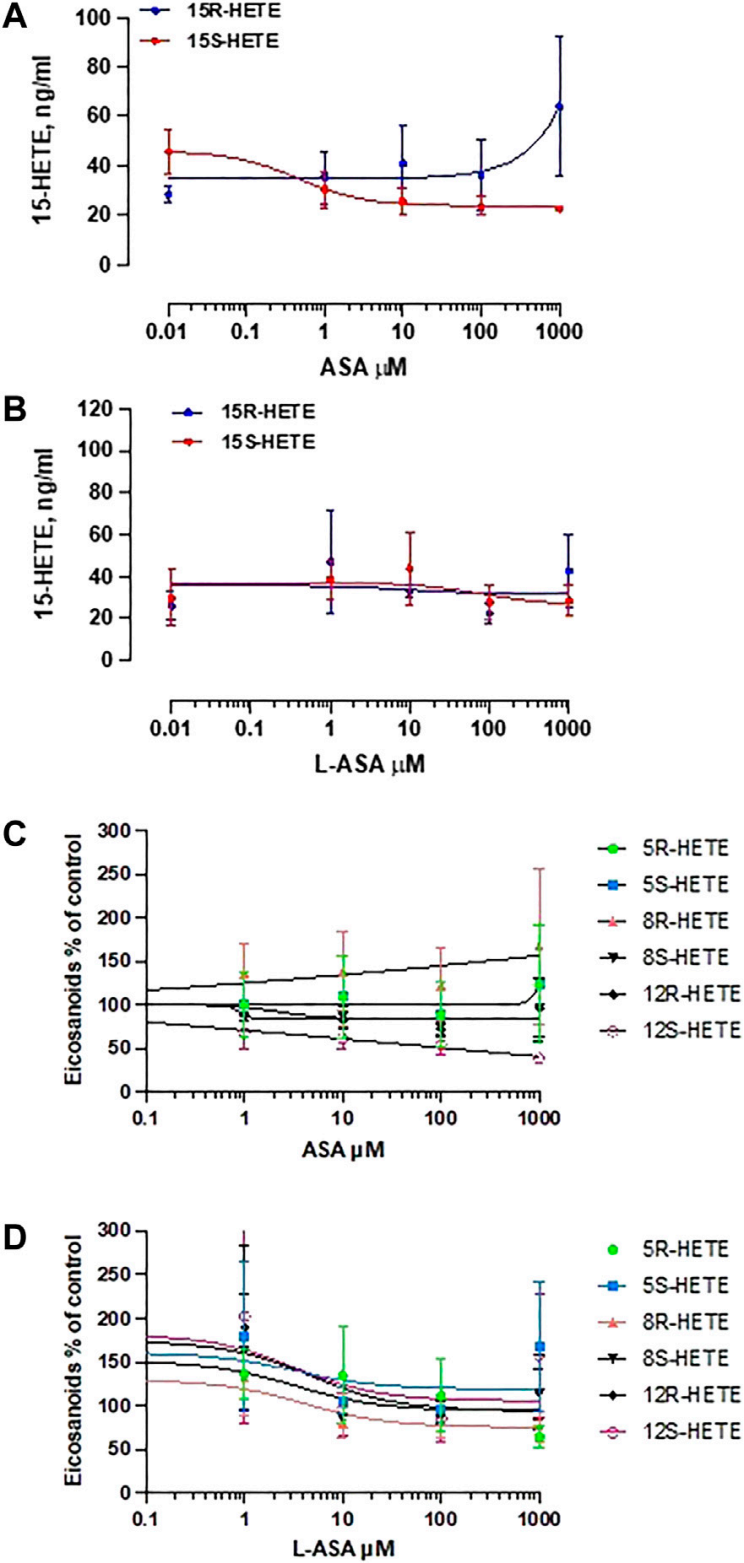
Effects of ASA and L-ASA on the generation of 15R-HETE and 15S-HETE and other HETEs in HCA7 cells in response to AA 100 μM. HCA7 cells were treated with DMSO or diluent or increasing concentrations of the ASA **(A)** or L-ASA **(B)** for 30 min at 37°C; then, cells were incubated with AA100 µM for 30 min at 37°C, and the levels of 15S-HETE and 15R-HETE were assessed by LC-MS/MS; data are shown as ng/ml and reported as mean ± SEM, n = 4. The effects of ASA and L-ASA on other HETEs is shown in panel **(C)** and **(D),** respectively and reported as % of control (mean ± SEM, n = 4).

Figure 8 shows the effect of ASA (panel A) and L-ASA (panel B) on 15R-HETE and 15S-HETE production by HCA7 cells stimulated with AA 100 μM. The two compounds caused variable and not significant effects. 15R-HETE was enhanced 2-fold, not significantly, only by ASA 1 mM, a supratherapeutic concentration (Figure 8A). 15-epi-LXA_4_ was undetectable in all conditions, although the cells presented acetylated COX-2 and active 5-LOX; in fact, 5S-HETE was detected in cell supernatant.

As shown in Figures 8C,D, ASA and L-ASA caused variable and marginal effects on the production of other HETEs.

## 4 Discussion

Several lines of evidence support the efficacy of low-dose aspirin (75–100 mg daily) in the secondary prevention of vascular events, such as stroke and myocardial infarction (Baigent et al., 2009). The clinical benefit of aspirin is thought to derive from the selective inhibition of TXA_2_ formed from AA in platelets by COX-1 (Patrignani & Patrono, 2015). Aspirin acetylates Serine529 of COX-1 (Picot et al., 1994), thus leading to irreversible inhibition of the enzyme associated with a persistent inhibition of platelet TXA_2_ biosynthesis (Patrignani et al., 1982; Patrignani et al., 2014). TXA_2_ is a potent vasoconstrictor and platelet agonist (Nakahata, 2008). Low-dose aspirin has to inhibit >97% serum TXB_2_ biosynthesis to cause an antiplatelet effect (Reilly & FitzGerald, 1987) because small residual concentrations of TXA_2_ can synergize with other platelet agonists to provoke complete platelet aggregation (Minuz et al., 2006). It has been proposed that aspirin’s antithrombotic efficacy may be limited by the coincidental inhibition of prostacyclin (PGI_2_), a major product of vascular endothelium *via* COX-2, exerting effects on platelet function and vascular tone opposite to those of TXA_2_ (Grosser et al., 2006). Thus, higher doses of aspirin are not recommended for chronic human treatment. This is also because higher doses of aspirin can be associated with an increased risk of gastrointestinal toxicity for the concurrent action of the inhibition of hemostasis and gastric damage (García Rodríguez et al., 2001; Patrignani et al., 2011; Patrignani & Patrono, 2015). Aspirin can cause surface injury that alters the gastric barrier to acid and the inhibition of COX-1-dependent PGE_2_, playing different cytoprotective effects. Thus, different formulations of aspirin have been developed to obtain a selective platelet COX-1 inhibition associated with reduced gastrointestinal toxicity. EC-asprin was developed to prevent aspirin’s direct effects on the gastric epithelial lining and to deliver aspirin to the intestine (Angiolillo et al., 2022). Some studies have reported that EC-aspirin leads to delayed and unpredictable absorption, particularly in younger and heavier patients and those with a previous myocardial infarction (Maree et al., 2005; Schneider, 2005; Cox et al., 2006; Grosser et al., 2013). However, we have found that after six consecutive low doses of EC-aspirin to healthy subjects, a virtually complete inhibition is obtained (for cumulative acetylation of platelet COX-1) that persisted throughout the dosing interval of 24 h (Patrignani et al., 2014). Although EC-aspirin did not substantially affect the systemic biosynthesis of PGI_2_, incomplete acetylation of colorectal COX-1 was associated with a roughly 50% reduction of intestinal PGE_2_ (Patrignani et al., 2017). Recently, a pharmaceutical lipid-aspirin complex (PL-ASA) was developed to mitigate disruption of the epithelial phospholipid layer of the gastric mucosa without delaying the absorption of aspirin (Angiolillo et al., 2022). Studies have shown that PL-ASA has similar bioavailability to rapid-release (plain) aspirin in fasted healthy volunteers and obese patients with diabetes. Moreover, PL-ASA significantly reduces the risk of acute gastric mucosal erosions and ulcers compared with immediate-release aspirin (Cryer et al., 2011). However, no information is available on the effect of this formulation on gastrointestinal COX-1-dependent PGE_2_ biosynthesis and the incidence of upper gastrointestinal bleeding. Another approach to maximizing the effect on the platelet and limiting the extraplatelet targets has been developed by a controlled-release preparation (Clarke et al., 1991; Charman et al., 1993). However, loading 162.5 mg of rapid-release aspirin (plain formulation, uncoated) to the regimen with a 75-mg dose of controlled-release aspirin was necessary to avoid the delayed inhibitory effect on platelet COX-1 with the controlled-release preparation (Clarke et al., 1991). The administration of this preparation was associated with small concentrations of ASA in the systemic circulation leading to limited extraplatelet effects (Clarke et al., 1991). Recently, the Food and Drug Administration (FDA) approved an extended-release aspirin formulation (durlaza, at 162 mg) for the secondary prevention of cardiovascular events and cardiovascular-related mortality (https://www.accessdata.fda.gov/drugsatfda_docs/nda/2015/200671Orig1s000TOC.cfm). Despite durlaza causing an approximately equivalent effect on TXB_2_ biosynthesis *ex vivo* and *in vivo versus* a rapid-release aspirin formulation of 81 mg, systemic exposure to aspirin was considerable (Patrick et al., 2015). Thus, this formulation can be associated with possible gastrointestinal side effects and reduced systemic biosynthesis of PGI_2_. However, this formulation could be helpful in patients with enhanced TXA_2_ biosynthesis from extraplatelet sources (Rade et al., 2022).

Here, we characterized the pharmacodynamics of a new aspirin formulation IP1867B (ASA/triacetin/saccharin), i.e., a stable liquid aspirin formulation. L-ASA can lead to enhanced cellular drug delivery due to triacetin, a water-soluble short-chain triglyceride. We found that L-ASA was more potent in inhibiting platelet COX-1 activity in human whole blood allowed to clot for 1 h at 37°C (serum) compared to ASA. Under these experimental conditions, TXB_2_ is a primary COX-1 product derived mainly from platelets (Patrono et al., 1980; Patrignani et al., 2014). This is an interesting finding since the assessment of this biomarker is used to evaluate the appropriate effect of aspirin as an antiplatelet agent (Patrono et al., 1980; Patrignani et al., 1982; Patrignani et al., 2014). We performed targeted chiral lipidomics to assess whether the two compounds influenced HETEs generated enzymatically and non-enzymatically in serum. Aspirin and L-ASA cause a concentration-dependent reduction of PGE_2_ and 15R-HETEs, which are minor AA products *via* COX-1 (Powell and Rokach, 2015); ASA resulted less potent than L-ASA to affect these eicosanoids. The other HETEs, including 12S-HETE, were not substantially affected. These data show that ASA and L-ASA specifically affect the COX one pathway in whole blood and that L-ASA resulted more potent than ASA.

We found that in the presence of the diluent of L-ASA (i.e., triacetin and saccharin), serum 12S-HETE levels were slightly higher than those detected in the presence of DMSO (the vehicle of ASA). All other serum eicosanoids were formed at comparable concentrations in the presence of the diluent or DMSO. Triacetin increases acetate bioavailability in different cell types *via* its hydrolysis to glycerol and acetate by non-specific lipases and esterases (Tsen et al., 2014). Enhanced AA can be produced in platelets in the presence of acetate accumulation by adding the two carbon atoms of acetate to the carboxyl group of exogenous plasma linoleic acid by removing four hydrogen atoms to form two additional double bonds. Interestingly our data suggest that this AA pool can be mainly metabolized by platelet 12-LOX. In platelets, 12S-HETE is mainly found esterified in six phospholipids, comprising four phosphatidylethanolamines and two phosphatidylcholines (Thomas et al., 2010; Contursi et al., 2021). The phosphatidylethanolamines include several plasmalogens representing oxidized forms of the most abundant phosphatidylethanolamine and phosphatidylcholine species in platelets. 12-HETE can be released from membrane phospholipids of platelets by the action of thrombin generated endogenously during whole blood clotting. Overall, this can lead to reduced levels of free 12-HpETE intracellularly. It is known that the extent of aspirin-dependent acetylation of platelet COX-1 is regulated by the enzyme’s catalytic activity of the peroxidase (Bala et al., 2008), which can also reduce 12-HpETE to 12-HETE. Thus, acetylation occurs most efficiently when the concentration of the hydroperoxide substrates (such as 12-HpETE) of COX peroxidase is low and is inhibited by high peroxide concentrations that generate the formation of the ferryloxo protoporphyrin radical cation (Bala et al., 2008). We propose that the more potent effect of L-ASA than ASA is influenced by the presence of triacetin which can modulate the generation of 12S-HpETE and its esterification of membrane phospholipids, thus promoting the capacity of ASA to acetylate COX-1. A specific study is necessary to address this hypothesis. However, this is supported by the finding that in the absence of plasma (i.e., washed platelets), ASA and L-ASA are equipotent to acetylate and inhibit platelet COX-1. However, the higher potency of L-ASA *versus* ASA in inhibiting serum TXB_2_ can also be explained by the influence of the diluent on ASA protein plasma binding or by the stabilization of ASA in plasma. Further studies are requested to address these points.

Low-dose aspirin has also been reported to reduce the risk of incidence and mortality for cancer, in particular, CRC (Patrignani & Patrono, 2016). The mechanism of aspirin action in this setting is still under debate. However, several lines of evidence suggest the contribution of the inhibition of platelet COX-1 by aspirin since platelets, once activated, can release numerous molecules, including TXA_2_, which can induce changes in cancer cell phenotypes and activate stromal cells to promote early events of tumorigenesis (Patrignani & Patrono 2016; Bruno et al., 2018; Bruno et al., 2022). However, it has been proposed that aspirin, even at low doses, can act by acetylating COX-2 at Serine516 in the epithelial, stroma, and cancer cells and that acetylated COX-2 cannot generate PGG_2_/PGH_2_ while producing 15R-HETE from AA (Lecompte et al., 1996). 15R-HETE could be transformed *via* 5-LOX to generate epimeric lipoxins, termed aspirin-triggered-LXs or 15-epi-LXs (Claria & Serhan 1995; Chiang et al., 2004; Serhan 2005). It has been hypothesized that the 15-epi-LXs, which are included in the family of specialized pro-resolving lipid mediators (SPMs), could underlie the antiinflammatory actions of aspirin. However, it has been recently reported that the capacity of tissues to form SPMs is very low *versus* traditional eicosanoids and other oxylipins. SPM levels in biological fluids would be at or below the limits of detection of the most sensitive MS instrumentation (Schebb et al., 2022). Here, we could not detect 15-epi-LXA_4_ in serum (the sensitivity of our assay was 10 pg/ml). Moreover, HCA7 cells treated with ASA or L-ASA and stimulated with high concentrations of AA showed acetylated COX-2 and generated 15R-HETE (mainly by the unacetylated COX-2 activity) and expressed a catalytic active 5-LOX, did not produce detectable 15-epi-LXA_4_ levels.

Our study shows that at more physiologic AA concentrations, HCA7 cells generated the COX products (PGE_2_ >> TXB_2_), and the different HETEs were almost undetectable. Thus, ASA and L-ASA act by affecting COX-2-dependent prostanoid biosynthesis. The finding that 15R-HETE production is involved in the effect of aspirin is not confirmed in HCA7 cells. First, at high AA concentration, 15R-HETE can derive from COX-2 activity. 15-LOX-1 could contribute to 15S-HETE production; in fact, we detected 12S-HETE, although HCA7 cells did not express platelet-type 12-LOX. 15-LOX-1 can metabolize AA to 15S-HETE and, to a minor extent, to 12S-HETE (Ivanov et al., 2015). Despite TXA_2_ being generated at lower concentrations than PGE_2_ in HCA7 cells, it is noteworthy that this prostanoid shows protumorigenic actions (Li et al., 2015).

In summary, ASA and L-ASA inhibited TXB_2_ biosynthesis and acetylated COX-1 at Serine529 in washed platelets with comparable potency. However, L-ASA was more potent in affecting serum TXB_2_ generation than ASA. The relevance of this finding remains to be evaluated *in vivo* in humans. In cancer cells, ASA and L-ASA act by inhibiting prostanoid generation associated with the acetylation of COX-2. ASA and L-ASA at clinically relevant concentrations did not substantially induce the biosynthesis of 15R-HETE and 15-epi-LXA_4_. 15R-HETE can be detected when non-physiological concentrations of AA and supratherapeutic concentrations of ASA are used, suggesting that it is not relevant *in vivo*.

In conclusion, we show that L-ASA is more potent in affecting platelet COX-1 than ASA in the presence of plasma. Whether this may also occur in tumor cells should be tested in animal models and patients after dosing with L-ASA *versus* ASA. Similarly to ASA, L-ASA acts by causing an irreversible inhibition of COX-1 and COX-2 *via* the acetylation at Serine529 and 516, respectively.

## Data Availability

The raw data supporting the conclusion of this article will be made available by the authors, without undue reservation.
