# Supplementary material for: Characterization of the acetylation of cyclooxygenase-isozymes and targeted lipidomics of eicosanoids in serum and colon cancer cells by the new aspirin formulation IP1867B versus aspirin in vitro
Source: Front Pharmacol. 2022 Dec 14;13:1070277. doi: 10.3389/fphar.2022.1070277 (PMC9795017; doi:10.3389/fphar.2022.1070277)
Supplement: Supplementary file 1 [file DataSheet1.docx]

## Supplementary Figures

**Supplementary Figure 1**. Baseline generation of eicosanoids in serum (whole blood allowed to clot for 1 h at 37°C) in the absence of vehicles and in the presence of DMSO or diluent (triacetin-saccharin). Values are shown as mean+SD, n=6. #P<0.05 *vs.* DMSO; *P<0.05 *vs.* no vehicle and DMSO using one-way ANOVA and Tukey multiple comparison test.

##

**Supplementary Figure 2.** Effects of increasing concentrations of ASA **(A)** and L-ASA **(B)** on eicosanoids generated in serum. Values are mean+SEM, n=3. Sigmoidal concentration-response curves of % inhibition data from baseline [in the presence of DMSO (ASA vehicle) or diluent (triacetin and saccharin, L-ASA vehicle)] were obtained by GraphPad Prism.

**A**

**B**


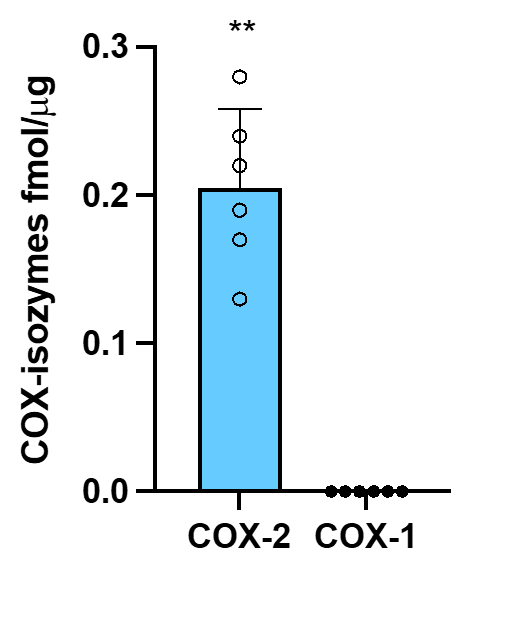

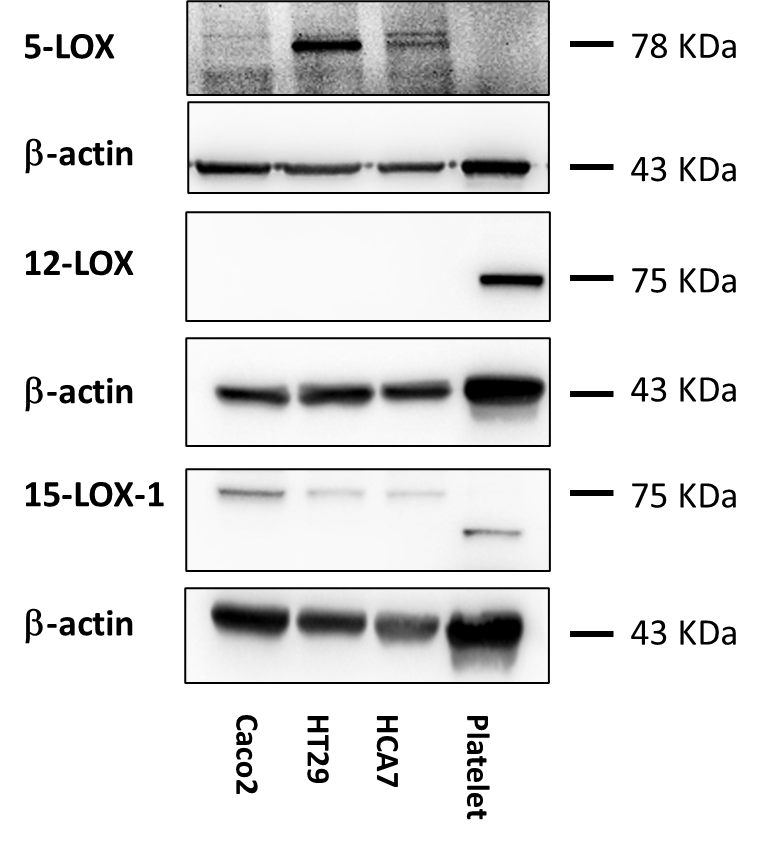


**Supplementary Figure 3. (A)**Baseline expression of COX-1 and COX-2 in HCA7 reported as fmol/µg of proteins, measured by LC-MS/MS. **(B**)Western blot analysis of 5-LOX, 12-LOX (platelet-type 12-LOX) and 15-LOX-1 expression in Caco2, HT29, HCA7 cells and platelets

**Supplementary Figure 4. Biosynthesis of eicosanoids by human colon cancer HCA7 cells in response to physiological or high concentrations of exogenous AA in the presence of DMSO or diluent (triacetin and saccharin) by targeted chiral lipidomics analysis using LC-MS/MS.** HCA7 cells (1 × 10^6^) were treated with DMSO or diluent (triacetin, saccharin) for 30 min, and then AA 0.5 **(A)**, 10 **(B)**, or 100 μM **(C)** was added, and the incubation was continued for further 30 min; then, prostanoids and HETEs were assessed in the conditioned medium by LC-MS/MS. Data are shown as mean+SEM (n=4) of eicosanoids.

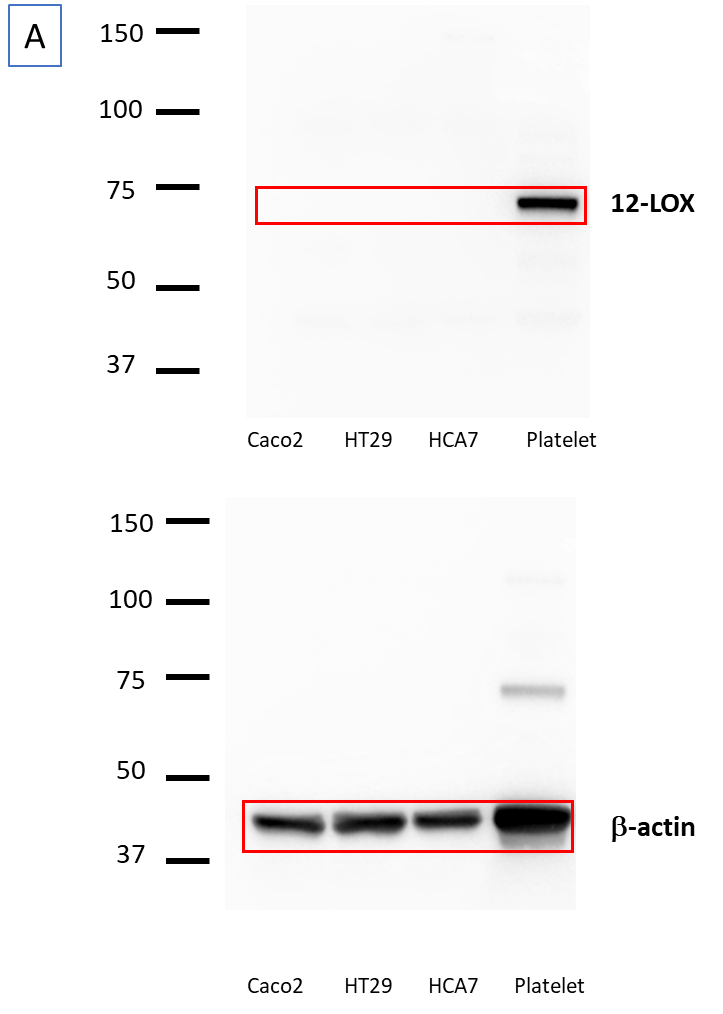

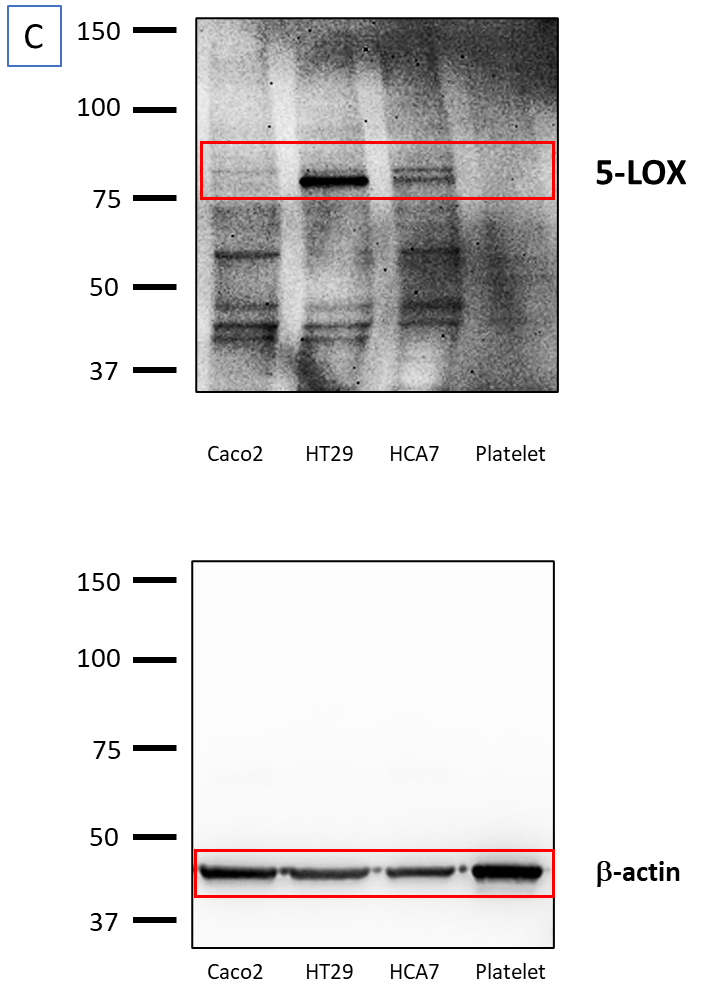

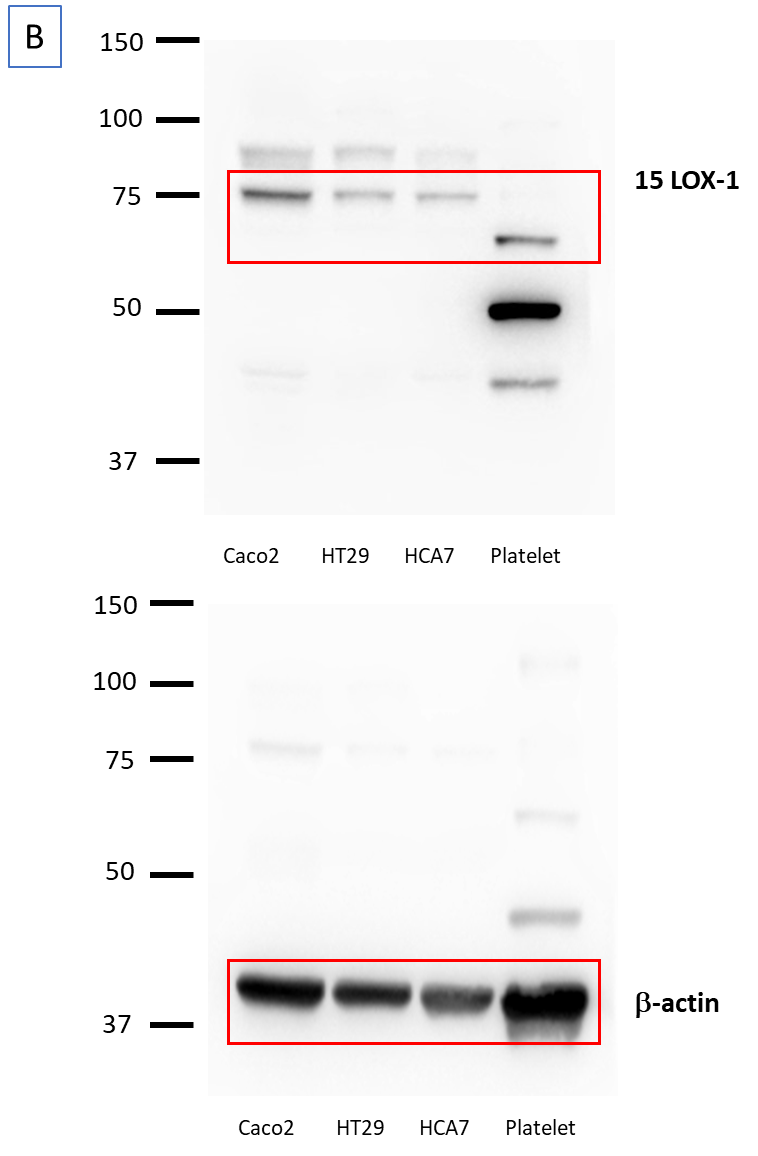


**Supplementary Figure 6.** Uncropped gel of western blotting membrane presented in Supplementary Figure 3B.

**Supplementary Table 1. Ions and selected product ions for each eicosanoid by LC-MS/MS and corresponding retention times**

**Supplementary Table 2. Inhibitory effects of ASA and L-ASA on serum PGE_2_ and 15R-HETE**

|  | **ASA** | **L-ASA** |
| --- | --- | --- |
| **PGE_2_** | 12.20(6.90-22.51) μM | 0.32(0.07-1.50) μM |
| **15R-HETE** | \| 10.07(6.43-23.44) μM \| \| --- \| | 0.76(0.07-6.00) μM |

Data are reported as IC_50_ and 95% confidence interval (into the parenthesis); they have been obtained by analyzing the sigmoidal concentration-response data of 3 separate experiments using GraphPad Prism software.

**Supplementary Table 3: Inhibitory effects of ASA and L-ASA on TXB_2_ and PGE_2_ generation in response to different concentrations of AA (0.5, 10, and 100μM) in HCA7 cells**

|  | **AA 0.5 μM** | **AA 10 μM** | **AA 100 μM** |
| --- | --- | --- | --- |
| **TXB_2_** | ASA: 10.72(5.85-20.00) μM  L-ASA: 12.78(7.60-22.00) μM | ASA: 8.66(6.25-12.01) μM  L-ASA: 7.98(3.50-17.48) μM | ASA: 6.83(3.85-12.09) μM  L-ASA: 7.04(3.15-15.30) μM |
| **PGE_2_** | ASA: 5.20(1.70-12.20) μM  L-ASA: 3.20(1.40-8.80) μM | ASA: 7.60(5.20-11.0) μM  L-ASA: 16.0(11.30-23.30) μM | ASA: 4.50 (3.10-6.60) μM  L-ASA: 7.50(4.10-13.80) μM |

Data are reported as IC_50_ and 95% confidence interval (into the parenthesis); they have been obtained by analysing the sigmoidal concentration-response data of 3 separate experiments using GraphPad Prism software.
